# Editorial: Neuromechanics and Control of Physical Behavior: From Experimental and Computational Formulations to Bio-inspired Technologies

**DOI:** 10.3389/fncom.2019.00013

**Published:** 2019-03-19

**Authors:** Manish Sreenivasa, Francisco J. Valero-Cuevas, Matthew Tresch, Yoshihiko Nakamura, Alfred C. Schouten, Massimo Sartori

**Affiliations:** ^1^Department of Mechanical, Material, Mechatronics and Biomedical Engineering, University of Wollongong, Wollongong, NSW, Australia; ^2^Division of Biokinesiology and Physical Therapy, Department of Biomedical Engineering, University of Southern California, Los Angeles, CA, United States; ^3^Shirley Ryan AbilityLab, Chicago, IL, United States; ^4^Department of Biomedical Engineering, Northwestern University, Evanston, IL, United States; ^5^Department of Mechano-Informatics, School of Information Science and Technology, University of Tokyo, Tokyo, Japan; ^6^Department of Biomechanical Engineering, Delft University of Technology, Delft, Netherlands; ^7^Department of Biomechanical Engineering, University of Twente, Enschede, Netherlands

**Keywords:** sensory-motor system, biorobotics, neuromechanical modeling, motor control, neuro-technologies, neural control of movement

## Introduction

The motivation behind this research topic was to cut across conventional boundaries that separate movement neuroscience, biomechanics, and robotics. The aim was to underscore that brain and body collaborate to produce behavior in biological organisms. While this is a simple idea, compartmentalization in education and science has often artificially separated brain from body. We also bring forward research paradigms to investigate physical behavior at the interface between humans and interacting robots. Understanding human-robot physical interaction requires the understanding of the complex interplay between brain, body, and the external environment. This could be achieved by employing a neuro-mechanical approach to the study of human and robot movement.

Within the context of a neuromechanical approach, we aimed to collate a research corpus that included work on experimental and neurophysiological analysis, computational modeling, and applications in rehabilitation and bio-inspired robotics. The 22 contributions to this research topic provide a wide range of perspectives and methodologies. The high-caliber contributions to this research topic also highlight the existence of a significant community of researchers interested in an interdisciplinary view toward the study of brain-body and human-robot interactions.

## The Contributions

Several of the studies enclosed in this research topic combined experimental work with state-of-the-art technologies and novel methodologies to measure and analyze neural control in humans (Banks et al.; Mojtahedi et al.; Logan et al.; Krüger et al.; Reyes et al.; Úbeda et al.).

A common theme among many studies was a cohesive and complimentary use of numerical modeling, signal processing and experimental approaches, with one supporting and enhancing the findings of the other to understand the basic mechanisms of movement (Guarín and Kearney; Golkar et al.; Song and Geyer; Petrič et al.; Laine et al.; Mehrabi et al.; Sreenivasa et al.; Alexandrov et al.; Lippi and Mergner; Pasma et al.; Von Walden et al.). Yet another inspiring aspect of this research topic was the crossover toward efforts in biomarkers of pathology and rehabilitation (Laine et al.; Reyes et al.; Sreenivasa et al.; Von Walden et al.; Shuman et al.; Banks et al.; Úbeda et al.) and robot control (Pasma et al.; Lippi and Mergner; Alexandrov et al.; Szczecinski et al.). Taken together, the contributions to this research topic illustrate the wide scope of research being conducted in neuromechanics and the vital role this will play in the future of rehabilitation and robotics.

The remainder of this editorial provides an overview of the scientific contributions. These are presented within three macro categories including:

1. Neuromechanics
2. Biotechnology and Rehabilitation
3. Bio-inspired Robotics

## Neuromechanics

Although there is detailed understanding of the mechanisms taking place in the central nervous system or in the musculoskeletal system during movement, there is far less knowledge of the interplay between these two systems during complex motor tasks (Tresch and Jindrich, 2014; Sartori et al., 2016, 2017; Sreenivasa et al., 2016; Valero-Cuevas and Santello, 2017; Cohn et al., 2018; Schouten and Mugge, 2019). This motivates a neuromechanical approach to the study of human behavior when producing movements and forces to interact with the environment.

One way to better understand the interplay between the neural and biomechanical apparatus is by identifying the system dynamics using a combination of models and experimental observations. Guarín and Kearney developed a methodology to dissociate the time-varying intrinsic and reflex components during movements at the human ankle joint. Their results indicate that the joint stiffness is modulated dynamically during non-stationary movements. The relevance of their work lies in proposing methodologies for extracting joint stiffness profiles during dynamic conditions, a step forward with respect to current methods that predominantly investigate quasi-stationary scenarios. Golkar et al. also focused on dynamic stiffness about the ankle joint, using human experiments to study the modulation of intrinsic and reflex stiffness with muscle activation. Logan et al. applied harmonic transfer function analysis to investigate how humans control upright posture and speed during locomotion. Their results support the existence of a temporal hierarchy of subtasks during locomotion, with the control upper-body posture taking precedence over others. Schumacher and Seyfarth used a feedback-based neuromuscular modeling approach to study reflex gains during hopping. Interestingly, their results indicate that different combinations of gain values enable optimizing specific hopping characteristics (e.g., performance and efficiency), and that this was invariant with respect to changes in the model's mechanical properties. With another feedback-based approach, Song and Geyer investigated the response of a walking model to simulated disturbances such as electrical stimulation and perturbations applied to the legs and to the whole-body. Their results show that the model behavior compares favorably to experimental recordings of similar disturbances during human locomotion. In a conclusion that resonates with this research topic, the authors comment on how such model approaches can complement the experimental study of human motor control.

Another branch of contributions investigated whole-body human movements (either free or when interacting with the environment), which require the coordination of multiple degrees of freedom, and are often directed toward completing specific tasks accurately and efficiently. The study of such movements provides the means to answer fundamental questions about motor control and brain-body interaction. Krüger et al. applied canonical correlation analysis to study movement variability in discrete goal-directed reaching tasks. They tested the effects of ischemia, (temporary) artificially reduced blood flow to the arms, and found that while this reduced the complexity of movement control, the endpoint variability did not increase. Petrič et al. comment on the lack of movement studies on real-world tasks such as hammering a nail into wood. In their work, they investigated the kinematics and dynamics of hammering both from an experimental and modeling perspective. They found that for periodic impact tasks, a model that relates the distance moved as a logarithmic function of time provided better predictions than Fitts' Law. Le Mouel and Brette proposed the hypothesis that postural adjustments follow spatial and temporal patterns that provide impetus for future movement. In a thought-provoking article, they relate this ability to learned proficiency in movements and the impairment of these skills with aging. Mojtahedi et al. studied how two humans collaborate to complete a physical task and the strategies used to infer movement direction. Interestingly, their findings suggest that the modulation of arm stiffness during cooperative tasks could be an effective means to communicate intended direction of movement. The authors relate this to future applications in human-robot interaction.

Even the simplest of movements often require the coordinated and smooth control of several muscles. Investigations at the neuromuscular level can provide an interesting way to reverse-engineer how the brain controls movements. Reyes et al. studied corticomuscular and intermuscular coherence between the muscles of the finger and the thumb during pinch grips. They found significantly reduced coherence when individuated control of the thumb and index finger was required, and interpret their findings as supporting the notion that the cortex bonds task-related motor neurons into task-dependent functional units. Laine et al. used complimentary experimental and modeling approaches to study how the neuromechanics of voluntary force production in the index finger were sufficient to produce involuntary tremor. The authors suggest that their findings represent a new category of tremor, one related to the viscoelastic response of a closed-loop musculotendon system to dynamic inputs. Moreover, they underscore the importance of coherence analysis, a well-established and neurophysiologically informative phenomenon (Farmer, 1998), to the study of muscle coordination in neuromechanics. Mehrabi et al. modeled a musculoskeletal planar arm and compared predictions using non-linear model predictive control (NMPC), to those from dynamic optimization and to experimental recordings. The authors motivate the NMPC approach by highlighting its effectiveness in simulating motion where the kinematics may or may not be prescribed, and where the target position may move during execution. Their results show that NMPC predicted the hand trajectory quite well, but not the hand velocities or muscle activations. Sreenivasa et al. developed optimal control-based predictive simulations of a child's gait with and without an ankle-foot orthosis. The motivation behind their work was to provide modeling-based tools to identify the patient-specific optimal stiffness of the orthosis, while reducing the effort required for the clinical procedure of fitting an orthosis to a patient. These studies inform the debate on the extent to which optimality (as understood by engineers) occurs in the neural control of our bodies (Loeb, 2012).

## Biotechnology and Rehabilitation

The knowledge gained from the study of human motor control, and the methods developed to analyze and synthesize human biomechanics are invaluable for the development of biomarkers of pathology, biotechnologies and rehabilitation processes that directly interact with the human body during complex movements. In a review article, Pizzolato et al. set an optimistic yet cautious note on the application of computational musculoskeletal models for the design and control of wearable devices. An interesting focus of the review was on bioinspired technologies that not only support or augment movement, but also foster tissue strengthening and repair through optimal tissue loading. Von Walden et al. studied the forearm flexor muscles in children with cerebral palsy (CP). They experimentally record the muscle strength and cross-sectional area, and use a modeling approach to estimate passive muscle stiffness independent of reflex activity. With a study title that conveys a concise take-home message, the authors found that the forearm flexor muscles of children with CP are weak, thin, and stiff, something that may inform personalized treatments. This underscores the plastic relationship between brain and body: damage to the neural controller can lead to physical changes in the anatomical actuators and plant.

The conclusions from Shuman et al. and Banks et al. are of significant importance to the research on muscle synergies during movements. Shuman et al. comment on the impact that filtering and scaling of recorded electromyography (EMG) data can have on the computed synergies for CP and typically-developing children, and the implications this can have when comparing results across studies and laboratories. Banks et al. investigated 30 variations in the methodology used for muscle synergy analysis, and evaluated the impact of these variations in identifying responders in post-stroke population. Their results highlight the sensitivity of MSA to methodological choices, and a need to standardize and/or provide exacting detail about the methodology used in future work. Úbeda et al. proposed a method to decode spinal primitives of multi-muscle control from electroenchephalography (EEG) recordings. The authors apply their method to healthy individuals as well as to patients with incomplete spinal cord injury, and comment on the future usage toward a new class of brain-exoskeleton interfaces. This work, together with that of Reyes et al. and Laine et al. helps clarify the ongoing debate on whether and how one can detect prescriptive synergies of neural origin—as opposed to descriptive synergies of numerical origin (Brock and Valero-Cuevas, 2016).

## Bio-inspired Robotics

Robot sensors and actuators remain vastly different from those on humans and animals, yet bio-inspired control methods find rich application in robotics. Szczecinski et al. developed methodology to design subnetworks that perform specific mathematical operations, and can be assembled into larger networks to mimic to some extent an animal's nervous system. Humanoid robots, with their anthropomorphic form and capabilities for bipedalism, are an excellent example for the translation of insights from human movement research to robotics. One interesting focus is on maintaining balance and posture, with robots possibly learning from humans, but also providing a real-world platform to test models and hypotheses for human balance. Alexandrov et al. proposed the use of the eigen-movement control concept derived from human hip-ankle coordination, as a simpler and more stable way to control humanoid robot balance. Lippi and Mergner implemented a modular human-derived control architecture for maintaining balance and posture in a humanoid robot. Interestingly, their results show that the mechanical coupling from the robot's body was sufficient to stabilize movements in the frontal and sagittal planes, without a need for an explicit link between the respective control modules. Pasma et al. quantified human balance control using the independent channel (IC) model and implemented the IC model in a humanoid robot. Their results show that the IC model, a descriptive model in the frequency domain, can imitate human balance behavior in real world situations with a humanoid robot. This provides further evidence that the IC model is a valid description of human balance control.

## Conclusions

This research topic posed direct focus on the neuromechanics of movements and the forces generated in interaction with the environment. This is described as an integrative approach that combines the neuromuscular control and the biomechanical aspects of body and the physics of the tasks in humans. We assert that this approach is overdue and necessary to obtain the theoretical and experimental frameworks for understanding the evolution, mechanics, neural control, energetics, disability, and rehabilitation of physical behavior. Moreover, understanding the neuromechanics of physical behavior should also leverage the development of personalized wearable robotic technologies that can interact with biological tissues within the composite neuromuscular system. This is central to ultimately mimic, restore or augment motor capabilities in healthy or impaired individuals.

## Author Contributions

All authors listed have made a substantial, direct and intellectual contribution to the work, and approved it for publication.

### Conflict of Interest Statement

The authors declare that the research was conducted in the absence of any commercial or financial relationships that could be construed as a potential conflict of interest.
